# Investigating the intensity of social contacts associated with tuberculosis: a weighted networks model

**DOI:** 10.1186/s12890-023-02519-z

**Published:** 2023-06-26

**Authors:** Neda Amoori, Payam Amini, Bahman Cheraghian, Seyed Mohammad Alavi

**Affiliations:** 1 Infectious and Tropical Diseases Research Center, Health Research Institute, Ahvaz Jundishapur University of Medical Sciences, Ahvaz, Iran; 2 Department of Biostatistics and Epidemiology, School of Public Health, Ahvaz Jundishapur University of Medical Sciences, Ahvaz, Iran

**Keywords:** Social Contacts, Tuberculosis, Weighted Networks Model

## Abstract

**Background:**

Tuberculosis is known as one of the principal health problems, especially in developing countries. This study aimed to visualize, statistically model, and describe the weighted networks to investigate the intensity of social contacts associated with tuberculosis.

**Methods:**

In this case–control study, we applied weighted network analysis to assess the network of person-time spent in stores, workplaces, restaurants, mosques, Police bases, homes, hospitals, colleges, hairdressers, schools, contact homes, health centers, cinemas, parks, and markets. Modules will be determined based on the similarities between the variables in a topology overlap matrix. The most important variables will be found considering the association between each variable and module eigenvalues.

**Results:**

The result shows the extracted modules of locations based on the connectivity followed by the person-time at each place. The correlation (*p*-value) between TB and the turquoise, blue, and brown modules was 0.058 (0.351), 0.004 (0.943), and 0.117 (0.039), respectively. The brown module is the most important one, demonstrating a significant connection between homes, contact homes, health centers, and hospitals. Therefore, an association was found between person-time in four places and the occurrence of TB.

**Conclusion:**

The finding of this study showed that most transmission of tuberculosis infection occurs in homes, contact homes, health centers, and hospitals. These place evaluations allow the identification of people with more contact and in need of screening, so critically leading to the identification of more patients with active TB.

## Introduction

The main cause of Tuberculosis(TB) is the bacterium Fastic acid Mycobacterium tuberculosis (MTB), and humans are the only reservoir [1, 2]. The disease is transmitted through the air from person to person and occurs worldwide. In 2020, the WHO estimated that there were 9.9 million contaminated with TB. This figure has been relatively constant in recent years. Tuberculosis caused 1.3 million deaths in 2020 and 1.2 million in 2019 worldwide [3]. Tuberculosis is treatable; however, it is among the top ten causes of death, and its "end" remains a remote reality [4]. In 2020, the incidence rates of this disease in the World, Eastern Mediterranean region and Iran were 132, 114 and 16 cases per 100,000 people, respectively [5].

Increasing susceptibility to MTB infection can be relevant to the following factors: co-morbidities like infection with the human infection virus(HIV), diabetes, silicosis or rheumatoid arthritis, and also other chronic illnesses or immunosuppressive therapies, and socioeconomic factors [6]. In addition, environmental factors such as people's lifestyle and gathering in crowded places can affect the risk of developing this disease [7].

Contact investigation is the procedure mainly used to identify more TB patients and those recently exposed to latent M. tuberculosis infection and considered at risk of progressing to TB. Studies show various amounts of success of TB-detection programs that elicited, located, and evaluated contacts of TB patients and started treatment for latent M. tuberculosis infection [8, 9]. Employing these programs to cover high-risk or vulnerable groups often shows limited success [10].

Social contact patterns influence the risk and epidemiology of airborne transmitted diseases such as MTB [11]. In addition to home contact, transmission of TB can occur during social contact outside the home and in social gatherings and due to exposure to infectious cases and susceptible community members [12].

To characterize TB contact patterns in prior social contact studies, only survey data and descriptive analyses have been used. Novel approaches to studying social contact patterns, such as employing weighted network analysis, were incorporated to expand on prior research [12]. Epidemiological studies often use contact networks to describe the interaction patterns within a population. Usually, these kinds of networks only show individuals who interact, but there is no indication of the strength or intensity of interactions [13].

In the present study, weighted networks, in which there is an associated weight for every connection, were used to examine the influence of heterogeneous contact strengths on the effectiveness of control measures. A way of building Weighted networks in shared community sites between those who respond and those unknown contacts is using data from venue-tracing interviews, which suggests a more thorough view of a community's Weighted networks [13]. Weighted networks and contact patterns are two factors that influence the spread and epidemiology of infectious diseases. We can analyze them to identify significant social network properties influential to the prevalence of MTB in community settings [14].This study aimed to visualize, statistically model, and describe the weighted networks to investigate the intensity of social contacts associated with tuberculosis.

## Subjects and methods

### Study population

This case–control study included 80 TB patients and 172 control participants. The selected cases were above 18-year-old pulmonary TB patients who were newly registered and bacteriologically confirmed. They referred to thirty-six health centers in Ahvaz, southwest Iran. The controls were attendees matched with the cases by age and sex, and their referral to the same health centers was due to non-TB health problems.

### Selection of cases

The cases were those who enrolled for treatment in the selected health centers in Ahvaz and were newly identified. The Sputum smear test and a complete medical evaluation for TB included chest radiology (X-rays), culture, medical history, physical examination, and tuberculin skin test were the primary tests for pulmonary TB. All newly registered TB patients were continuously included in the data collection process until reaching the required sample size. There were two selected controls for each case.

### Selection of controls

Attendee in the same facility with the age (within 5-year age bands) and sex, matched with an individual case, was selected as control. Those who met the age and sex criteria were investigated on a clinic day. Those who met the age and sex criteria were studied on a clinic day. If the attendee refused to participate in the study, the next clinically eligible attendee would be examined. To manage the controls' medical complaints and perform a clinical screening to exclude pulmonary TB, the study doctor visited them. If the person referred to the clinic had any sign of suffering from TB, the necessary TB laboratory tests would be performed according to the national diagnostic algorithm. AFB microscopy tests are provided free of charge, so they were not charged for that. However, the study covered the cost of other probable investigations like X-rays.

### Data collection

First, the research sample was selected based on the inclusion criteria. After elaborating on the purposes of the study to the participants, they were assured of the confidentiality of the information collected, and consent informed was obtained from all of them. Then using checklists, standard forms, and face-to-face interviews information was collected. which includes: demographic variables, social-economic and environmental factors.The present study examined all social contacts of the case and control groups during the month before they entered the study. All participants reported these main places where there was social contact: a private home, contact home, Police base, workplace, mosque, school, market and shop, college, restaurant, cinema, hairdressers, park, hospital, and health center. For each reported social contact during the previous month, participants were asked to express the number of times on average per week that they had contact (e.g., daily, twice, three times) and also the average duration of each contact in hours. For every reported place and social contact, person-time was calculated. The assessment was performed by multiplying the average weekly frequency of each contact by the average duration of each contact. For example, if a participant reports meeting a social contact two times a week for 2.5 h per meeting, the contact time would be 5 h per week. This study is a part of the thesis results and the other part is published in another journal [15].

### Statistical analysis

The descriptive characteristics of continuous and categorical variables are shown in mean (standard deviation) and frequency (percentage), respectively. Independent samples t-test is used to compare the mean value of continuous variables between case and control groups. The Chi-square test was utilized to evaluate the association between categorical variables and group variables.

To assess the network among of person-time spent in areas such as stores, workplaces, restaurants, mosques, Police bases, homes, hospitals, colleges, hairdressers, schools, contact's homes, health centers, cinemas, parks, and markets we used weighted network analysis approach [16]. We applied the weighted gene co-expression network analysis using the R package WGCNA in R programming software [16].To find the best power for the subsequent analysis of the network, the pickSoftThreshold function and 90% cut-off point for scale-free topology model fit. The resulting power contributes to constructing the adjacency matrix. Later, the TOM similarity function was used to calculate the topological overlap matrix and the corresponding dissimilarity from the adjacency matrix. Hierarchical clustering using the average method was applied to the dissimilarity matrix and the first principle component of each clustering module was calculated to form the module eigenvalue (ME). The heat map of the sample expression was plotted using the resulting MEs and the module with the highest value of person-time was determined as the key module. The association between the modules and the presence of tuberculosis, the MEs, and person-time spent at each area in modules was calculated using correlation tests and the intramodular connectivity function. The resulting statistics in this step contributed to finding the hub area in the key modules. The constructed network was analyzed through the cytoHubba plugin under Cytoscape software V 3.9 [17].

## Results

A total of 80 cases and 172 controls participated in the study. The mean age of patients was 34.1 years (15.3%) and also for the control group was 32.5 years (12.0%). As shown in Table 1., more than half of the cases (77.5%) and controls (51.7%) were males. In the case group, about 30% were single and 61.3% were married, and in the control group, 27.3% were single, and 65.7% were married. Two-thirds of the cases(63.8%) and 20.9% of the controls had primary education, of which about 47.5% and 43% of the controls had a formal job. 27.5% of the cases and 5.2% of the controls were underweight.

**Table 1:** Patients’ characteristics in the case and control groups

Variables		**Case(n=80)**	**Control(172)**	***P*-value**
**Age, mean (sd)**		34.1(15.3)	32.5(12.0)	0.08
**Sex, n(%)**	Male	62(77.5)	89(51.7)	<0.001
	Female	18(22.5)	83(48.3)	
**BMI, n(%)**	Underweight	22(27.5)	9(5.2)	<0.001
	Normal	45(56.3)	114(66.3)	
	Overweight	9(11.3)	30(17.4)	
	Obese	4(5.0)	19(11.0)	
**Marital status, n(%)**	Single	24(30.0)	47(27.3)	0.62
	Married	49(61.3)	113(65.7)	
	Divorced	3(3.8)	10(5.8)	
	Widowed	4(5.0)	2(1.2)	<0.001
**Educational status, n(%)**	Illiterate	16(20)	2(1.2)	
	Read and Write	13(16.3)	3(1.7)	
	Up to elementary	23(28.7)	31(18.0)	
	Secondary school	15(18.7)	47(27.3)	
	College or more	13(16.3)	89(51.7)	
**Employment Status, n(%)**	Employed	38(47.5)	74(43.0)	<0.001
	Unemployed	42(52.5)	98(57.0)	
**Size of the family, n(%)**	< 2	25(31.2)	21(12.2)	<0.001
	2-4	48(60.0)	133(77.3)	
	>4	7(8.8)	18(10.5)	
**Monthly Income, n(%)**	Less than 100 dollars	24(30.0)	21(12.2)	0.04
	100-200 dollars	26(32.5)	59(34.4)	
	200-300 dollars	18(22.5)	32(18.6)	
	More than 300 dollars	12(15.0)	60(34.9)	

The median household size for the case and control groups was 2 and more than half of the cases (60.0%) and controls (77.3%) had less than four household members. In 32.5% of cases, the monthly income was between $ 100 and 200 and 34.9% of the controls lived on a monthly income of more than $ 300.

**Fig. 1:**
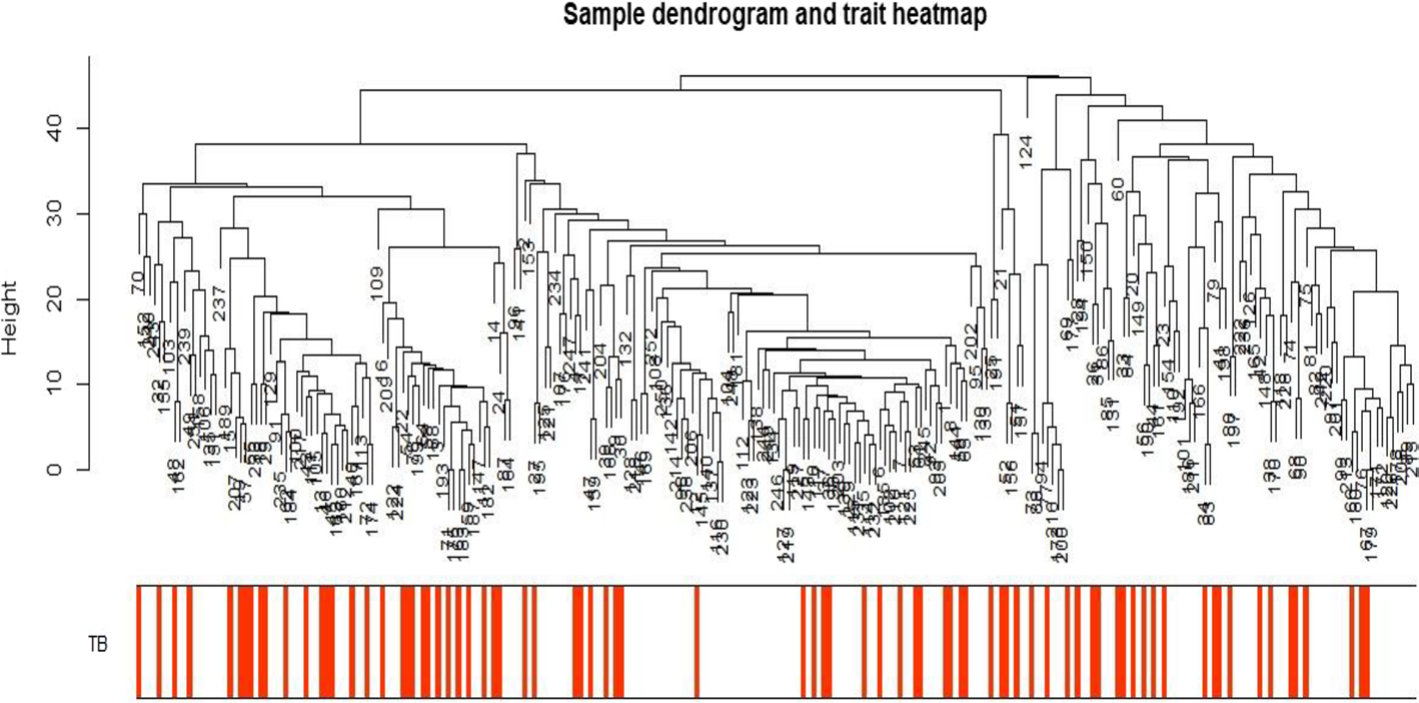
Sample dendrogram showing the potential extreme values and the association between TB and locations

A total of 3778 unique locations have been reported. The cases reported 1510, and the control group reported 2268 unique locations. First, we assess the presence of extreme values as well as the association between TB and locations by plotting the sample dendrogram (Fig. 1). White means low value and grey means high value of the relationship for each sample.

**Fig. 2:**
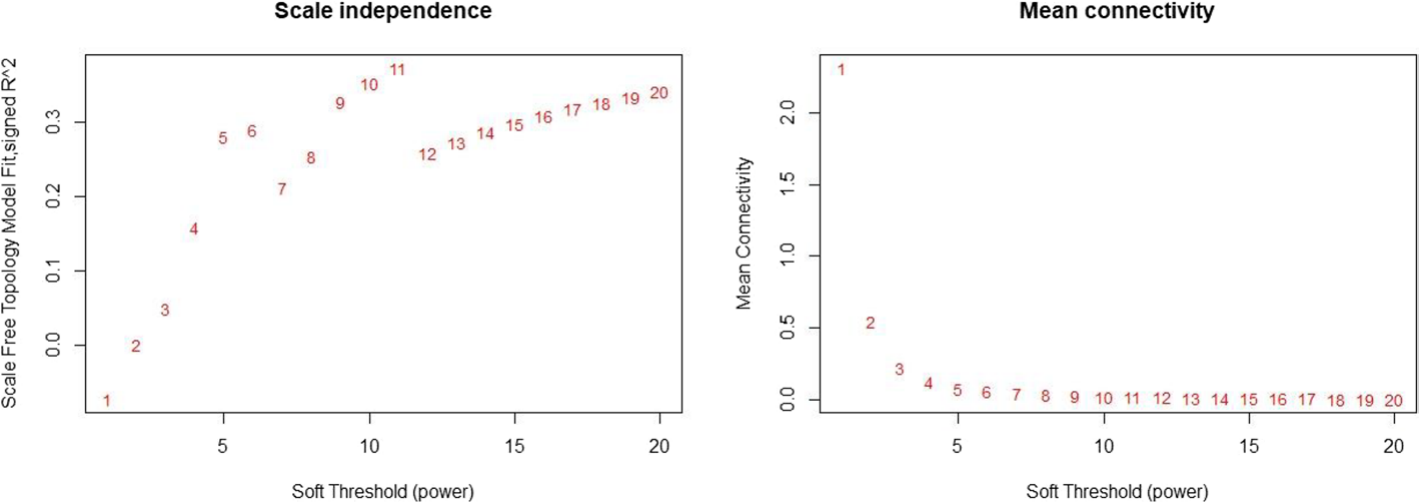
Finding the best power for the adjacency matrix of locations based on scale-free topology model fit index and mean connectivity

**Fig. 3:**
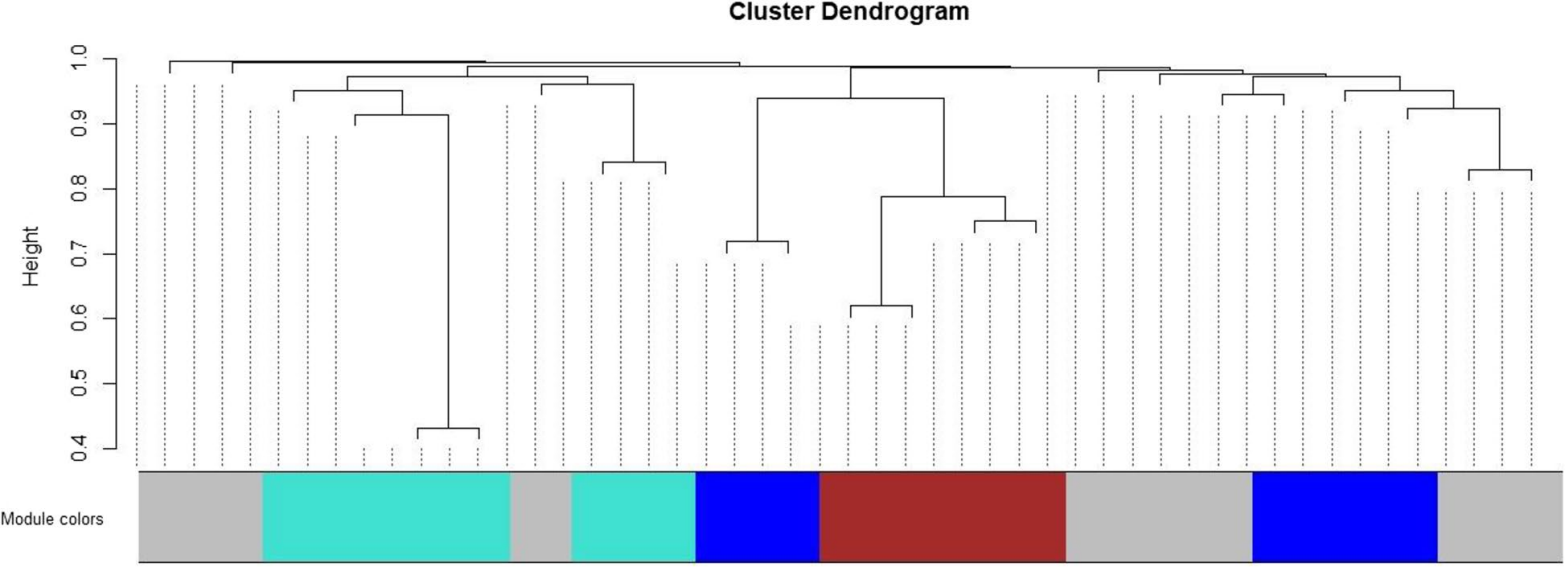
Finding modules based on the connectivity regarding the person-time spent at each location

To create a neighborhood matrix, it is necessary to select the neighborhood function. Here, considering the weight of the network, we use the power function. Based on Fig. 2, one can observe a set of powers and the best value for the power function parameter of the neighborhood can be chosen using the number with the highest R^2^ index. In this data, we consider the value of 5 as network power. Due to the small number of variables, low R^2^ is normal. Figure 3 shows the result of extracted modules of locations based on the connectivity followed by the person-time at each location. Stores, workplaces, hairdressers, schools, parks, and markets are in the turquoise module. The blue module contains a restaurant, mosque, Police base, college, and Cinema. Brown module is formed by locations including homes, hospitals, contact homes, and health centers. Eight locations in the grey module are not classified in any of the resulting important modules regarding their low correlations.

The most important module can be found regarding its association with TB. The correlation (p-value) between TB and the turquoise module is 0.058(0.351), the blue module is 0.004(0.943), and the brown module is 0.117(0.039). The intensity of association between the brown module and TB is highest among the resulting modules. It is highly important to be noticed that Pearson's correlation test evaluates the intensity of association and the magnitude of the coefficient is not interpretable (Table 2).

**Table 2:** Comparison of the relationship between tuberculosis disease and different modules

Module	Correlation with TB	P
MEturquoise	0.058899	0.351773
MEblue	0.004451	0.943954
MEbrown	**0.117659**	**0.039186**
MEgrey	0.124842	0.047738

**Fig. 4:**
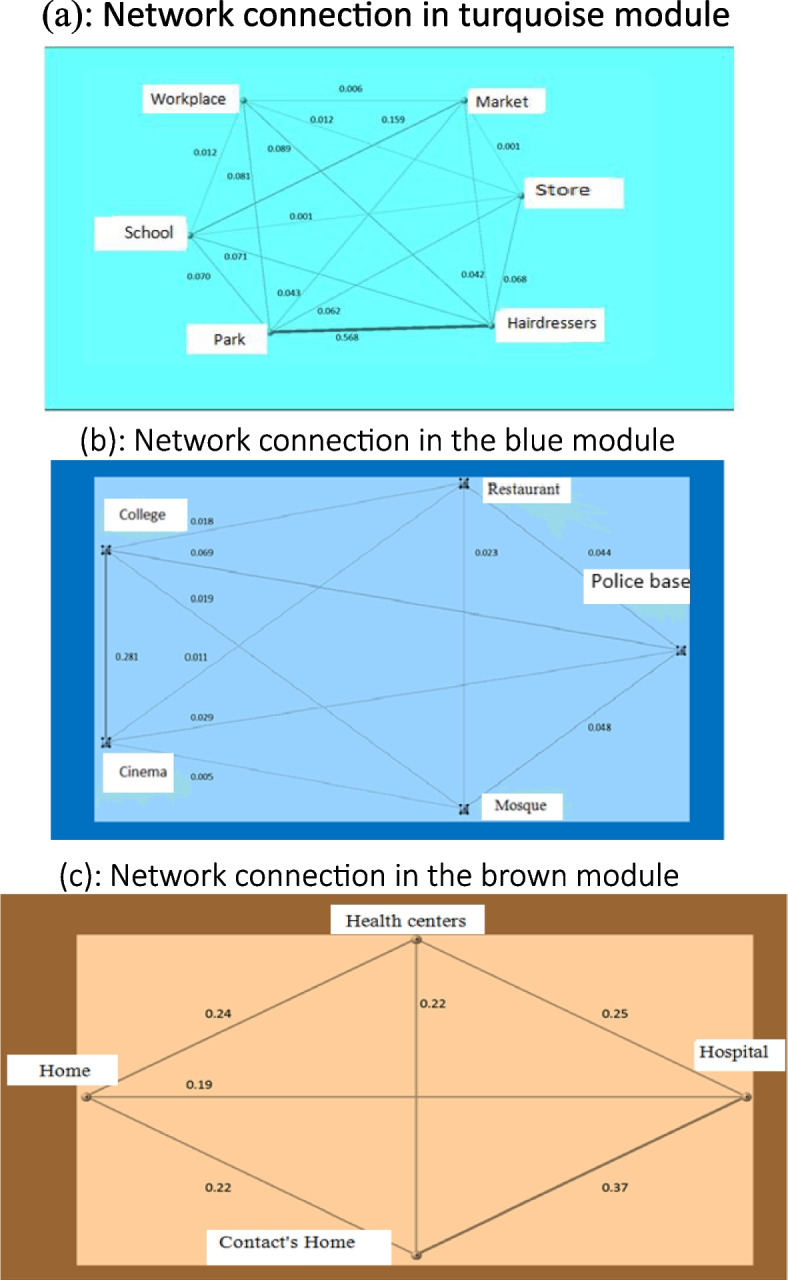
**a** Network connection in turquoise module. **b** Network connection in the blue module. **c** Network connection in the brown module

The final network for each module is shown in Fig. 4a-c. In the following three diagrams, the relationship between the variables of each module based on the communication weight is given. The weight takes a value between 0 and 1, and a higher value means more weight for the relationship between the variables. Among the modules, the brown module is the most important module in which different places such as home, contact home, health center, and hospital have a significant connection with each other. The amount of connectivity related to the home with the health center, contact home and the hospital is 0.25, 0.37, and 0.19, respectively, which shows that the home has a stronger connection with the rest, that is, this module shows that these 4 variables have a very significant and more closely related connection (Fig. 4c). In the blue and turquoise modules, different places such as restaurants, Police bases, mosques, etc. are related to each other, but the amount of communication is close to zero. In the module, it is the same (Fig. 4a, b). Finally, it is shown that the most important variable in this network is home, and contact home, which has a very strong relationship with each other. Therefore, in the brown module, it was found that the person-time in 4 places including home, contact home, health center, and the hospital is related to the occurrence of tuberculosis, and tuberculosis itself was affected by this module. Of the 4 places mentioned, home and contact home are closely related to the others.

## Discussion

This study aimed to visualize, statistically model, and describe the weighted networks of MTB cases to identify MTB-related spatial social contact processes. A total of 252 people participated in this case–control study conducted in Ahvaz, Iran, which included 80 pulmonary tuberculosis patients confirmed in terms of bacteria, and 172 controls.

In this study, weight networks related to the effect of 15 different locations on tuberculosis based on person time were examined. Network connections were examined in 3 modules: turquoise, blue, and brown. In these modules, variables of places were evaluated.

In the blue (Corr = 0.004, *P* = 0.94) and turquoise (Corr = 0.058, *P* = 0.35) modules, different locations were related, but their association with the disease was about zero. Among these modules, the brown module(Corr = 0.117, *P* = 0.039) had the highest rate of association with tuberculosis, in which various locations, including home, contact home, health center, and hospital, had very close and significant connections with each other and their association with tuberculosis was the highest.

The finding of this study shows that most transmission of tuberculosis infection occurs at home and in contact home. The results of the present study were similar to the previous studies [18]. Contact tracking is considered to be effective in identifying individuals who have been infected recently and it has become an essential component of TB eradication strategies in most countries with low prevalence. Inevitably, not all contacts are detected or screened. The reasons for this are low level of education, fear of stigma, lack of counseling, the unwillingness of index patients to identify all the people in contact with him or her, and lack of motivation of some people to go to the center for screening [19].

Furthermore, according to the results of this study, in addition to home contacts, many contacts occur in non-home environments such as health centers and hospitals, which indicates the risk of transmission in the community [20, 21]. These healthcare facilities are the first connection points where there is a risk of TB infection, and a strong infection control system is needed in these areas. Our results are consistent with other studies which recognized healthcare settings as high-risk environments for TB transmission [22]. Also, other studies have shown that visiting social sites improves the likelihood of discovering an unclear epidemiological link between patients [23]. Visiting locations associated with the transmission is recommended by The US National Tuberculosis Controllers Association and the US Centers for Disease Control and Prevention because site visits can add contacts who otherwise would not be identified [24].

Despite following standard procedures during interviews with TB patients, and attempting to list the names of contacts(within each transmission site) and the approximate type, frequency, and duration of exposure comprehensively, additional contacts may always be found, because the index patients do not always remember their contacts or simply do not want to name them. Contact research, while consuming financial resources, is an essential component of a successful National Tuberculosis Program (NTPs). Therefore utilizing an efficient strategy for contact investigation is a priority for public health [25, 26].

### Strength of the study

In other studies, in which this model has been used, only how the infection spreads in infectious diseases has been explained in general. These studies show that the physical interaction network within the home and workplace and the network of all physical interactions are considered two kinds of social mixing that might influence disease spread. The survey was performed in two settings, and may not be representative of the whole population [22–24, 26]. To complete the previous studies, the present study utilized the weighted networks model to investigate the influence of different social environments, in addition to home and workplace, on tuberculosis. This information allows planning for interventions to maximize the use of rare resources or workforce.

### Limitations of the study

The degree of goodness of fit in this model was equal to 30%, which may be due to the small number of variables in this study.

## Conclusions

In summary, this study provides, weight networks related to the effect of 15 different locations on tuberculosis based on person time. Network connections of different locations of this disease were investigated in three modules: turquoise, brown, and blue. From among these modules, the brown module had the highest rate of association with tuberculosis, in which various locations, including home, contact home, health center, and hospital, had very close and significant connections with each other and their association with tuberculosis was the highest. According to the results of the study, a revision of the program for preventing the spread of tuberculosis is needed.

## Data Availability

Data is available upon request to the corresponding author.

## Abbreviations

MTB: Mycobacterium tuberculosis
HIV: Human infection virus
ME: Module eigenvalue
